# Evaluation of the Diurnal Cycle of Blood Pressure and Sleep in Shift Workers

**DOI:** 10.7759/cureus.48029

**Published:** 2023-10-31

**Authors:** Divya Gupta, Latika Mohan, Arun Goel, Rajesh Kathrotia

**Affiliations:** 1 Physiology, All India Institute of Medical Sciences, Rishikesh, Rishikesh, IND; 2 Physiology, Indira Gandhi Institute of Medical Sciences, Patna, IND; 3 Physiology, All India Institute of Medical Sciences, Rajkot, Rajkot, IND

**Keywords:** sleep pattern, ambulatory blood pressure, blood pressure monitoring, diurnal, circadian, shift work tolerance

## Abstract

Background: Circadian misalignment of physiological factors in shift workers is poorly studied in the Indian population. In the present study, 24-hour blood pressure measurements were taken on the same subject twice, once during his morning and night shifts. Sleep was also monitored by a self-reported sleep diary, which was confirmed with an activity monitor, and the sleep quality was assessed using sleep questionnaires.

Objective: This study aimed to discover the pattern of blood pressure variation, the dipping and non-dipping status, and its correlation with sleep.

Methodology: This observational study was conducted in the Department of Physiology, All India Institute of Medical Sciences (AIIMS), Rishikesh, from April 2019 to September 2019, among security guards working rotating shifts in the Rishikesh hospital premises. Participants were given an activity sheet with instructions to document their daily activities for a complete 24-hour period on the designated measurement day, including recording the time of waking up and going to sleep. A wrist-worn activity monitor was utilised to assess the self-reported sleep duration provided by each participant on the activity sheet.

Results: The present study showed the mean age of the participants as 27.03 ± 2.71 years, along with a mean body mass index (BMI) of 22.10 ± 1.64. Sleep duration was significantly higher during the morning shift (5.81 ± 1.08 hours) compared to the night shift (4.02 ± 1.70 hours) on the day of ambulatory blood pressure monitoring (ABPM) recording. The mean difference in systolic blood pressure between night shift workers between their awake and sleep periods was 15.91 ± 8.44 mmHg. However, no statistically significant disparity was seen when comparing the systolic blood pressure at the 24-hour mark during wakefulness and sleep between those working morning and night shifts (p >0.05).

Conclusion: The current study's findings indicate that participation in shift work, particularly night shift work, could potentially play a role in the emergence of irregular circadian blood pressure patterns and potentially lead to a lack of nocturnal blood pressure decline.

## Introduction

Individuals who work in shifts are more prone to developing cardiovascular disease compared to those who work typical daytime hours [1]. Individuals who engage in shift work exhibit a 17% higher probability of encountering any cardiovascular disease event in comparison to those who adhere to a regular daytime work schedule [2]. Moreover, individuals engaged in shift work encounter a significantly elevated risk of myocardial infarction, with a 23% increase in comparison to individuals working regular daytime hours [2]. There is an elevated occurrence of hypertension among shift workers in comparison to persons who adhere to conventional daytime working hours, such as the typical 9 a.m. to 5 p.m. schedule [3]. Furthermore, individuals who are employed on night shifts are at a significantly higher risk of hospitalisation due to coronary artery disease [2]. One plausible hypothesis could be the repetitive and prolonged exposure to elevated blood pressure that is experienced throughout work shifts [4]. The theory indicated above is supported by empirical research that demonstrates a clear association between the duration of shift work engagement and the likelihood of developing cardiovascular disease [5]. The work schedule of shift employees is characterised by their regular involvement in non-traditional working hours, which diverge from the conventional daylight timetable of 9 a.m. to 5 p.m. [6]. Furthermore, it is common for these individuals to experience prolonged bouts of wakefulness that might persist for 12 hours, 24 hours, or even more [6]. The practice of shift employment involves the regular rotation of predetermined work hours, with transitions between daytime, evening and nighttime shifts. The work pattern indicated above is linked to the occurrence of both acute and chronic sleep deprivation, as well as a deterioration in sleep quality [4].

The impact of sleep and circadian variation is substantial in the diurnal rhythm of blood pressure [7]. The circadian rhythm of blood pressure is commonly typified by elevated levels of both systolic and diastolic blood pressure at awakening, followed by sustained blood pressure levels throughout the day. Subsequently, a reduction of roughly 10-20% in blood pressure occurs throughout the nocturnal and sleep phases [7]. Shift work, specifically night shift employment, has been found to alter the regular diurnal blood pressure cycle [8]. The nocturnal work schedule has a disruptive effect on the typical association between sleep and circadian rhythms, resulting in alterations in blood pressure variability. This phenomenon primarily arises due to the requirement for nocturnal shift workers to be alert during nighttime hours and rest during daylight hours [9]. The phenomenon of lower blood pressure during sleep, known as the "dip," is shown to be less pronounced in specific populations of individuals who work night shifts, such as shift workers [9-10]. The presence of diminished nocturnal blood pressure decline and/or reduced blood pressure during sleep has been linked to an elevated risk of stroke, target organ damage, left ventricular hypertrophy, impaired renal function, and cardiovascular disease-related mortality [11-12]. The probable correlation between shift work, sleep deprivation, and the disturbance of typical blood pressure patterns has been recognised as a noteworthy contributing factor in understanding the heightened vulnerability to cardiovascular disease among individuals involved in shift work [8].

A wide range of research has provided a comprehensive understanding of the intricate and contradictory relationship between shift work, blood pressure, and the probability of developing cardiovascular disease (CVD) [8]. Shift work is an exemplary event that perturbs the conventional 24-hour circadian rhythm of blood pressure oscillations and has a detrimental impact on blood pressure levels during the sleep period [10]. Based on several research, it has been noted that individuals who work night shifts experience sustained elevation in BP that persists beyond the initial 12-24 hours of relaxation and sleep following their work shift [13-14]. However, alternative studies suggest that the impact on blood pressure is insignificant. The temporary nature of the possible impact of shift work on blood pressure may be attributed to individuals adapting to the timing and scheduling of their shifts [15-16].

Therefore, to strengthen the scientific evidence regarding circadian misalignment in shift workers, the present study was planned. Moreover, physiological mechanisms underlying the observed clinical effects remain poorly understood. Therefore, we have attempted to study the pattern of circadian BP variation and sleep in Indian shift workers so that it might help in understanding the pathophysiology.

Aim and objectives

The purpose of this study was to determine the diurnal variation of blood pressure and sleep in relation to shift employment. The primary objective of this study was to measure blood pressure and heart rate continuously throughout the day and night shifts for drawing comparison blood pressure variation during the day versus the night shifts. Furthermore, this study also focuses on evaluating the associations between sleep time and blood pressure.

## Materials and methods

This observational study was conducted in the Department of Physiology, All India Institute of Medical Science (AIIMS), Rishikesh, from April 2019 to September 2019 to evaluate the association of sleep patterns with blood pressure parameters among security guards working in shifts in the Rishikesh hospital. The study was conducted after attaining ethical approval from the Institutional Ethics Committee, AIIMS Rishikesh (AIIMS/IEC/18/492). The study's inclusion criteria involved normotensive security guards within the age range of 18-45 years. Meanwhile, all the security guards having thyroid disorders, coronary artery disease, hypertension, any sleep disorders or consuming any medications were excluded from the research. 

Sample size calculation

The sample size was computed by the McNemar test exactly with an odd ratio of 6 and a discordant proportion of 0.5. The overall sample size of 30 was computed through the test with the power of 80%; however, a total of 36 participants was recruited with an error margin of 5%. 

Outcome parameters

The primary outcomes of the study encompassed multiple parameters measured during a 24-hour period, namely the average blood pressure (both systolic and diastolic mmHg), the percentage decrease in blood pressure during nighttime hours relative to daytime hours, the average heart rate, the total duration of sleep, and the quality of sleep. 

An ambulatory blood pressure monitoring (ABPM) device (TM-2430, A&D Company, Tokyo, Japan) was used to record the 24-hour blood pressure values at fixed 30-minute intervals. An activity monitor watch, sleep diary (activity sheet), and two sleep questionnaires, BSWSQ (Bergen Shift Work Sleep Questionnaire) and ESS (Epworth Sleepiness Scale), were used for assessing sleep. 

The recruited participants were working in rotating shifts, namely morning, evening, and night shifts, each lasting for one week. Subjects were informed one day before the study to come to the department at the specified time; then, their informed written consent was taken after explaining the whole procedure verbally. The demographic characteristics, including height, weight, and resting blood pressure, were measured for each participant. 

Participants were given an activity sheet with instructions to document their daily activities for a complete 24-hour period on the designated measurement day. This included recording the time of waking up and going to sleep. A wrist-worn activity monitor was utilised to assess the self-reported sleep duration provided by each participant on the activity sheet. The reason this technology was used is because of the empirical finding that individuals exhibit reduced physical activity during sleep and increased physical activity during wakefulness. The monitors have accelerometers, movement detectors, and ample memory capacity to store data for many weeks. The act of capturing and storing movement data occurs at several intervals within a single second to conduct subsequent analysis at a later time.

Sleep questionnaires were used to assess the quality of sleep. On the subsequent day, sleep patterns were assessed without ambulatory blood pressure monitoring (ABPM) to investigate the potential impact of the ABPM equipment on sleep quality.

The ambulatory blood pressure monitoring device was affixed to the patients for a continuous duration of 24 hours and, after that, retrieved on the following day. Participants were instructed to maintain immobility and refrain from arm movement while the device was active. The same protocol was adhered to during their night shifts.

The majority of the recordings were conducted between April and August to mitigate the impact of temperature fluctuations on the investigation and to prevent exposure to high temperatures.

During the period of ambulation, the ABPM device's memory was utilised to take measurements, which were subsequently transmitted to a computer. All collected data were then recorded in a computer file. The researchers monitored the duration of sleep and then gathered other outcome measures.

The paired student t-test was utilised to compare the outcome variables between morning and night shifts. A significance level of less than 0.05 was used to determine statistical significance.

## Results

The observations and results produced by the present study are compiled and displayed in the form of tables and charts. Table 1 illustrates the basal clinical characteristics of the subjects. The mean age was observed as 27.03 ± 2.71. At baseline, the average resting systolic blood pressure was observed as 115.70 ± 3.62, while the mean resting diastolic blood pressure was reported as 76.44 ± 3.73 (mmHg).

**Table 1:** The fundamental clinical characteristics of the individuals. SBP: systolic BP; DBP: diastolic BP

	Mean	SD
Age (years)	27.03	2.71
Weight (kg)	64.11	4.46
Height (cm)	170.33	2.67
BMI (kg/m^2^)	22.10	1.64
Resting SBP (mmHg)	115.70	3.62
Resting DBP (mmHg)	76.44	3.73
Resting PR (beats per min)	78.22	3.23

In Table 2, a detailed analysis shows the comparison of sleep parameters and blood pressure parameters of morning shift and night shift workers. We compared the different outcome parameters like 24-hour mean systolic blood pressure, diastolic blood pressure, initial mean arterial blood pressure (MABP), pulse pressure, pulse rate, and sleep between morning and night shifts of the same subject. Reduction in systolic blood pressure was observed in awake and sleep periods among morning and night shift workers; however, insignificant statistical differences were observed when comparing the 24-hour mean (p=0.876), awake (p=0.125) and sleep (p=0.448) SBP during morning and night shifts. The mean systolic blood pressure of 21.41 ± 10.95 mmHg was observed during the awake-sleep period among the morning shift workers while 15.91 ± 8.44 mmHg systolic blood pressure was observed during the awake-sleep time of night shift workers (p-value <0.024). As a result, the percentage dipping of SBP was also significantly different between morning and night shifts. Percentage dipping was 15.28 ± 7.56% during the morning shift and 11.56 ± 5.95% during the night shift (p-value<0.05)

**Table 2:** Comparison of mean and standard deviation of various outcome parameters between morning and night shifts SBP: systolic blood pressure; DBP: diastolic BP; PP: pulse pressure; MABP: mean arterial BP; ESS: Epworth Sleepiness Scale

Parameters		Morning shift		Night shift		Paired t-test
		Mean	SD	Mean	SD	P-value
SBP (mmHg)	24hr	133.2	11.55	132.86	11.11	0.876
	Awake	139.42	12.1	135.69	11.73	0.125
	Sleep	118.01	13.89	119.78	10.93	0.448
	Awake-sleep	21.41	10.95	15.91	8.44	0.024
	Avg dipping (%)	15.28	7.56	11.56	5.95	0.024
DBP (mmHg)	24hr	80.01	6.67	80.84	7.03	0.468
	Awake	84.88	7.35	83.46	7.37	0.324
	Sleep	68.05	8.74	69.56	8.51	0.35
	Awake-sleep	16.84	8.11	13.91	8.17	0.188
PP (mmHg)	24hr	53.19	8.89	52.02	8.41	0.451
	Awake	54.54	8.87	52.23	8.69	0.188
	Sleep	49.96	10.34	50.23	9.8	0.885
	Awake-sleep	4.58	6.83	2.01	8.46	0.196
MABP (mmHg)	24hr	97.4	7.51	97.83	7.63	0.755
	Awake	102.72	8.18	100.53	8.07	0.18
	Sleep	84.38	9.58	85.93	8.19	0.358
	Awake-sleep	18.34	8.58	14.61	7.25	0.075
Pulse rate (beats/min)	24hr	77.1	6.32	76.65	8.01	0.683
	Awake	81.71	7.01	78.77	8.46	0.025
	Sleep	65.02	7.52	68.29	9.66	0.014
	Awake-sleep	16.7	7.12	10.48	7.57	0.000
Sleep duration (hours)	Day of recording	5.81	1.08	4.02	1.7	0.000
	Next day	5.78	1.01	4	1.54	0.000
	ESS score	6	1.24	14.04	1.84	0.000
	ESS score	13.86	1.85	14.8	1.79	0.33

**Figure 1:**
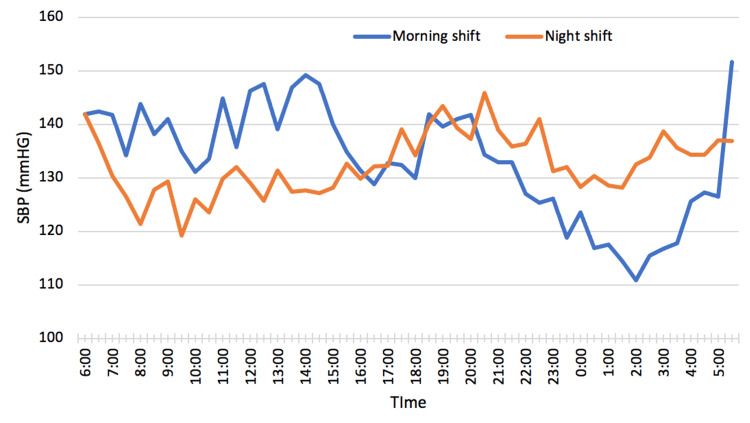
Variation of SBP in a 24-hour cycle during morning and night shift SBP: Systolic BP

Figure 1 depicts the graphical representation of the fluctuation in the mean systolic blood pressure (SBP) during a 24-hour period for all participants, separately for the morning and night shifts. The blue line represents the morning shift, while the orange line represents the night shift.

Table 3 illustrates the difference between sleep duration on the day of ABPM recording and on the next day without ABPM recording. Sleep duration was significantly higher during the morning shift (5.81 ± 1.08 hours) as compared to the night shift (4.02 ± 1.70 hours) on the day of ABPM recording. This suggests that sleep is reduced in workers during their night shifts which may affect their health.

**Table 3:** Comparison of sleep duration on the day of ABPM recording and on the next day of recording without ABPM ABMP: Ambulatory blood pressure monitoring

	Morning Mean ± SD	Night Mean ± SD	p-value
Duration of sleep on the day of recording (in hours)	5.81 ± 1.08	4.02 ± 1.70	0.68
Duration of sleep on subsequent dat (in hours)	5.78 ± 1.01	4.00 ± 1.54	0.87

During the night shift, sleeping patterns were different among subjects. The majority of subjects (N=22) were morning sleepers (sleeping before 12 PM) and the remaining (N=5) were afternoon sleepers (sleeping after 12 PM). Table 4 illustrates the difference in systolic blood pressure, diastolic blood pressure, pulse pressure, mean arterial pressure, pulse rate, sleep duration, and ESS score during the night shift between those who sleep before 12 p.m. and after 12 p.m.

**Table 4:** Comparison of various outcome parameters between morning and afternoon sleepers during their night shift SBP: systolic blood pressure; DBP: diastolic BP; PP: pulse pressure; MABP: mean arterial BP; ESS: Epworth Sleepiness Scale

Parameters		Sleep time before 12 PM (N=22)		Sleep time after 12 PM (N=5)		Paired t-test
		Mean	SD	Mean	SD	P value
SBP (mmHg)	24hr	131.36	11.78	139.47	2.51	0.01
	Awake	134.07	12.37	142.84	3.5	0.01
	Sleep	118.7	11.25	124.55	8.75	0.24
	Awake-sleep	15.37	8.39	18.29	9.18	0.54
	Avg dipping (%)	11.28	5.95	12.77	6.48	0.66
DBP (mmHg)	24hr	79.82	7	85.33	5.76	0.11
	Awake	82.22	7.03	88.94	6.9	0.1
	Sleep	69.42	8.8	70.16	8.01	0.86
	Awake-sleep	12.8	7.55	18.78	9.91	0.26
PP (mmHg)	24hr	51.54	8.93	54.14	5.78	0.44
	Awake	51.85	9.36	53.9	5.19	0.52
	Sleep	49.28	9.84	54.4	9.48	0.32
	Awake-sleep	2.57	8.94	-0.49	6.02	0.38
MABP (mmHg)	24hr	96.65	7.81	103.02	4.13	0.03
	Awake	99.16	8.02	106.56	5.51	0.04
	Sleep	85.46	8.53	87.96	6.91	0.51
	Awake-sleep	13.7	6.66	18.6	9.21	0.31
Pulse rate (beats/min)	24hr	76.69	8.64	76.51	4.97	0.95
	Awake	78.72	9.13	78.99	5.24	0.93
	Sleep	68.6	10.07	66.93	8.44	0.71
	Awake-sleep	10.12	7.83	12.05	6.83	0.6
Sleep duration (hrs)	Day of recording	4.11	1.84	3.6	0.89	0.38
	Next day	4.09	1.64	3.8	0.91	0.6
	ESS score	13.86	1.85	14.8	1.79	0.33
	Awake-sleep	10.12	7.83	12.05	6.83	0.6
Sleep duration (hrs)	Day of recording	4.11	1.84	3.6	0.89	0.38
	Next day	4.09	1.64	3.8	0.91	0.6
	ESS score	13.86	1.85	14.8	1.79	0.33

Table 5 illustrates the assessment of sleep quality done using the BSWSQ. Median scores were calculated for each of the seven parameters during different shifts and also on rest days. Analysis of the Bergen Shift Work Sleep Questionnaire indicates that shift workers report more sleep disturbance and impaired wake-time performance during the night shift. 

**Table 5:** The median score during different shift times and rest days Basic Shift Work Sleep Questionnaire (0-4 scoring); 0=Never, 1=rarely, 2=sometimes, 3=often, 4=always M: morning; E: evening; N: night

Basic Shift Work Sleep Questionnaire (BSWSQ)	Shift time			
	M	E	N	Rest days
Sleep onset latency is defined as the duration of time above 30 minutes required to initiate sleep.	1	2	3	1
The phenomenon of wakefulness after sleep onset refers to instances of wakefulness that occur for a duration exceeding 30 minutes during the primary sleep period.	0	2	3	1
The phenomenon of premature awakening refers to the occurrence of waking up more than 30 minutes earlier than desired, without being able to fall back asleep.	1	0	2	0
Experiencing insufficient restfulness subsequent to sleep.	0	0	3	0
The phenomenon of experiencing fatigue or drowsiness during work hours	0	1	3	-
The phenomenon of experiencing fatigue or drowsiness during unoccupied periods on workdays.	1	1	3	-
The phenomenon of experiencing fatigue or drowsiness during periods of non-work or vacation.				0

Table 6 illustrates the association of total sleep duration during both morning and night shifts with dipping percentages of SBP, mean SBP, DBP, and pulse pressure. A negative correlation between sleep duration with dipping percentages during the night shift can be explained by the fact that blood pressure varies according to the stage of sleep.

**Table 6:** Association of duration sleep with dipping percentages of mean SBP, DBP, and pulse rate DBP: diastolic blood pressure; SBP: systolic blood pressure

Parameters	Morning shift	p-value	Night shift	p-value
	Duration of sleep correlation coefficient r		Duration of sleep correlation coefficient r	
% Dipping	0.07	0.71	-0.492	0.009
SBP	-0.218	0.27	-0.07	0.72
DBP	-0.157	0.43	-0.14	0.489
Pulse rate	0.147	0.46	0.102	0.61

## Discussion

Shift work refers to a form of employment that extends beyond the standard eight-hour working period, hence disrupting the inherent circadian rhythm of the human body. Individuals who work night shifts are more susceptible to cardiovascular disease than those who work during regular daytime hours [1]. The nocturnal nature of shift work poses a larger threat to overall health due to its direct influence on the body's biochemical and physiological systems [17]. Shift work has experienced a rise in popularity as a scheduling strategy to efficiently deploy a limited workforce to ensure continuous operations. The existing body of literature provides evidence that employees encounter several challenges, including heightened stress levels connected to their employment, inadequate sleep, nicotine use, unhealthy eating habits, and disruptions in their sleep-wake patterns [18]. These particular lifestyles can elicit disturbances in the typical metabolic and hormonal processes of the human body, leading to subsequent physiological stress and alterations in the circadian rhythm.

The present study showed the mean age of the participants as 27.03 ± 2.71 years, along with a mean body mass index (BMI) of 22.10 ± 1.64. The findings of our study are inconsistent with the research conducted by Kouchaki et al. [19] since they observed a higher BMI among shift workers compared to day workers, even after accounting for potential confounding variables. Similarly, Marqueze et al. [20] arrived at the same conclusion, namely that night shift workers experience a more pronounced increase in BMI and weight gain compared to their counterparts who work during the day. Our study aligns with the findings of Zamanian and colleagues [21]. It was revealed that there is no significant alteration in body mass index (BMI) among individuals employed in shift work compared to those working during the day. 

In the present investigation, there was no statistically significant disparity seen when comparing the systolic blood pressure at the 24-hour mark throughout times of wakefulness and sleep between those working morning and night shifts. Nevertheless, a marginal elevation in systolic blood pressure was noted in both groups throughout the wakefulness and sleep phases. The mean difference in systolic blood pressure between night shift workers between their awake and sleep periods was 15.91 ± 8.44 mmHg. The findings align with the systematic review by Patterson et al. [22], wherein they observed that the average systolic blood pressure among shift workers was higher. This conclusion was based on an analysis of 11 research papers, which revealed a mean difference of 18.48 mmHg (95% confidence interval: 16.71 to 20.26). According to a systematic review conducted by Gamboa et al. [23] it was found that constant night employment was associated with the highest estimate of a 2.52 mmHg increase in systolic blood pressure. This estimate had a 95% confidence interval ranging from 0.75 to 4.29. The evaluation included 12 research with 29,923 participants, and there was a high degree of heterogeneity (I2 = 91%) among the included studies. 

The phenomenon characterized by the absence of a decrease in blood pressure during nighttime, commonly referred to as non-dipping, has been found to be linked to metabolic and cardiovascular disorders. In the current study, the percentage dipping was 15.28 ± 7.56 (%) during the morning shift and 11.56 ± 5.95 (%) during the night shift (p value<0.05). Our study revealed that the dipping percentage was more during the morning shift and less during the night shift. These results are consistent with the study of Karelius et al. [24] in which they found more SBP dipping among night shift workers than regular workers (14% vs 16%). Meanwhile, Toffoli et al. [25] found a significant decrease in SBP dipping percentage from 12.6% to 1.49% among night shift workers.

During the monitoring phase, we evaluated the sleep duration for each security guard for several days using a sleep questionnaire. It was noticed that the duration of sleep was considerably greater during the morning shift (5.81 ± 1.08 hours) in comparison to the night shift (4.02 ±1.70 hours) on the day when ambulatory blood pressure monitoring (ABPM) was conducted. The observed result indicates decreased sleep duration among workers during night shifts, potentially impacting their overall health. The findings presented align with the research conducted by Fatima et al. [26], who determined that night shift employees already experience insufficient rest activity, which significantly impacts their health. Inadequate rest directly contributes to an increase in catecholamine levels, hence disrupting normal blood pressure regulation. The reduction in nocturnal sleep duration can be ascribed to various variables, including an irregular sleep pattern and sleep initiation during atypical circadian phases [27]. The phenomenon observed during nocturnal periods is characterised by a reduction in sleep duration and an elevated level of arousal towards the latter portion of the disrupted sleep cycle. This phenomenon frequently arises when individuals are assigned to work on the night shift without having undergone proper circadian adjustment [27]. 

The present investigation observed that the average pulse rate of those working during the night shift was comparatively lower than that of individuals working during the morning shift (76.65 ± 8.01 vs 77.10 ± 6.32 beats per minute, respectively). The findings presented in this study align with the outcomes reported in the prior investigation conducted by Hussain and colleagues [28]. Nicoletti et al. [29] conducted an observation wherein they found that the average rise in heart rate during night shifts was significantly lower compared to day shifts (p < 0.01). Hansen et al. [30] conducted a study investigating the impact of night shifts on blood pressure and heart rate. The researchers observed that after seven days, there was no discernible alteration in these physiological parameters. Based on their findings, the authors postulated that the primary factor influencing blood pressure and heart rate during night shifts might be the heightened activity level at night, or the circadian drive may not substantially influence these cardiovascular measures.

The findings of this study demonstrate that individuals who work shifts experience greater levels of sleep disruption and decreased performance upon waking during their night shifts, as evidenced by the analysis of the BSWSQ. The increased prevalence of sleep disturbances during night shifts can be related to the disruption of the circadian rhythm and the inherent preference for nocturnal sleep. Additionally, individuals generally exhibit improved cognitive performance during daytime hours. Singh and Kwatra [31] observed a notable alteration in physiological indicators, including heart rate and blood pressure, which indicate stress and tiredness resulting from the demands of cyclic job tasks.

In the current investigation, an additional objective was to examine the potential relationship between sleep duration and various cardiovascular parameters, including the percentage decrease in systolic blood pressure, the average systolic blood pressure, the diastolic blood pressure, and the pulse rate. However, no statistically significant correlations were observed, except for a negative correlation between sleep duration and the percentage decrease in systolic blood pressure, specifically during night shifts. The p-value was found to be less than 0.05. An inverse connection was seen between inadequate sleep duration and the percentage of individuals experiencing drowsiness among night shift employees. The observed inverse relationship between sleep duration and the percentage change in blood pressure among individuals working night shifts can be attributed to the fluctuation of blood pressure during different stages of sleep. The blood pressure levels exhibit their lowest values during the deep stages of sleep, specifically stages 3 and 4. Blood pressure levels exhibit an elevation during the stages of sleep characterised by reduced depth, namely stages 1, 2, and rapid eye moment sleep [31-33].

Limitations of study

This study has certain limitations. Persuading participants to engage in continuous 24-hour ambulatory blood pressure monitoring is a challenging task. Ambulatory blood pressure monitoring is not commonly employed as a routine practice in India, thus making it a novel concept for the general populace. Certain individuals exhibit apprehension over utilising the apparatus despite receiving counselling. Occasionally, the subjects' family members experience fear, leading to the necessity of removing the machine. Due to its high cost, the provision of this system to OPD patients or the general population may pose significant challenges. The quantity of ambulatory blood pressure monitoring machines constrains the number of subjects that can be investigated simultaneously.

## Conclusions

The current study's findings indicate that participation in shift work, particularly night shift work, could potentially play a role in the emergence of irregular circadian blood pressure patterns and potentially lead to a lack of nocturnal blood pressure decline. Furthermore, it can potentially impact both the duration and quality of sleep. Further investigation is required to ascertain the factors contributing to the daily blood pressure fluctuations. Additional research is needed to investigate the impact of shift work on individuals, particularly those of Indian descent. Limited attention has been given to studying the effects of shift work, specifically on Indian shift workers. It is crucial to include race as a significant determinant in understanding the diurnal variation of blood pressure.
